# P-1375. Clinical Profiles and Treatment Outcomes of Indian Children with Drug-resistant Central Nervous System Tuberculosis

**DOI:** 10.1093/ofid/ofaf695.1562

**Published:** 2026-01-11

**Authors:** Dhruv Gandhi, Viren Amesur, Minnie Bodhanwala, Ira Shah

**Affiliations:** Bai Jerbai Wadia Hospital for Children, Mumbai, India, West Monroe, LA; Bai Jerbai Wadia Hospital for Children, Mumbai, India, West Monroe, LA; Wadia Group of Hospitals, Mumbai, India, Mumbai, Maharashtra, India; Bai Jerbai Wadia Hospital for Children, Mumbai, India, West Monroe, LA

## Abstract

**Background:**

Pediatric central nervous system tuberculosis (CNS-TB) accounts for 10% of all pediatric TB cases in India and carries the highest morbidity and mortality. However, data on pediatric drug resistant (DR) CNS-TB remains scarce. Regional data from Mumbai suggests that pediatric DR CNS-TB accounts for about 12% of all pediatric DR-TB cases. This dearth of epidemiological data translates to a lack of clinical studies on pediatric DR CNS-TB. This study aims to address this knowledge gap by analyzing the clinico-demographic profiles, laboratory findings, treatment regimens and outcomes, and adverse drug reactions (ADR) in a cohort of Indian children diagnosed with DR CNS-TB.

**Table 1:**
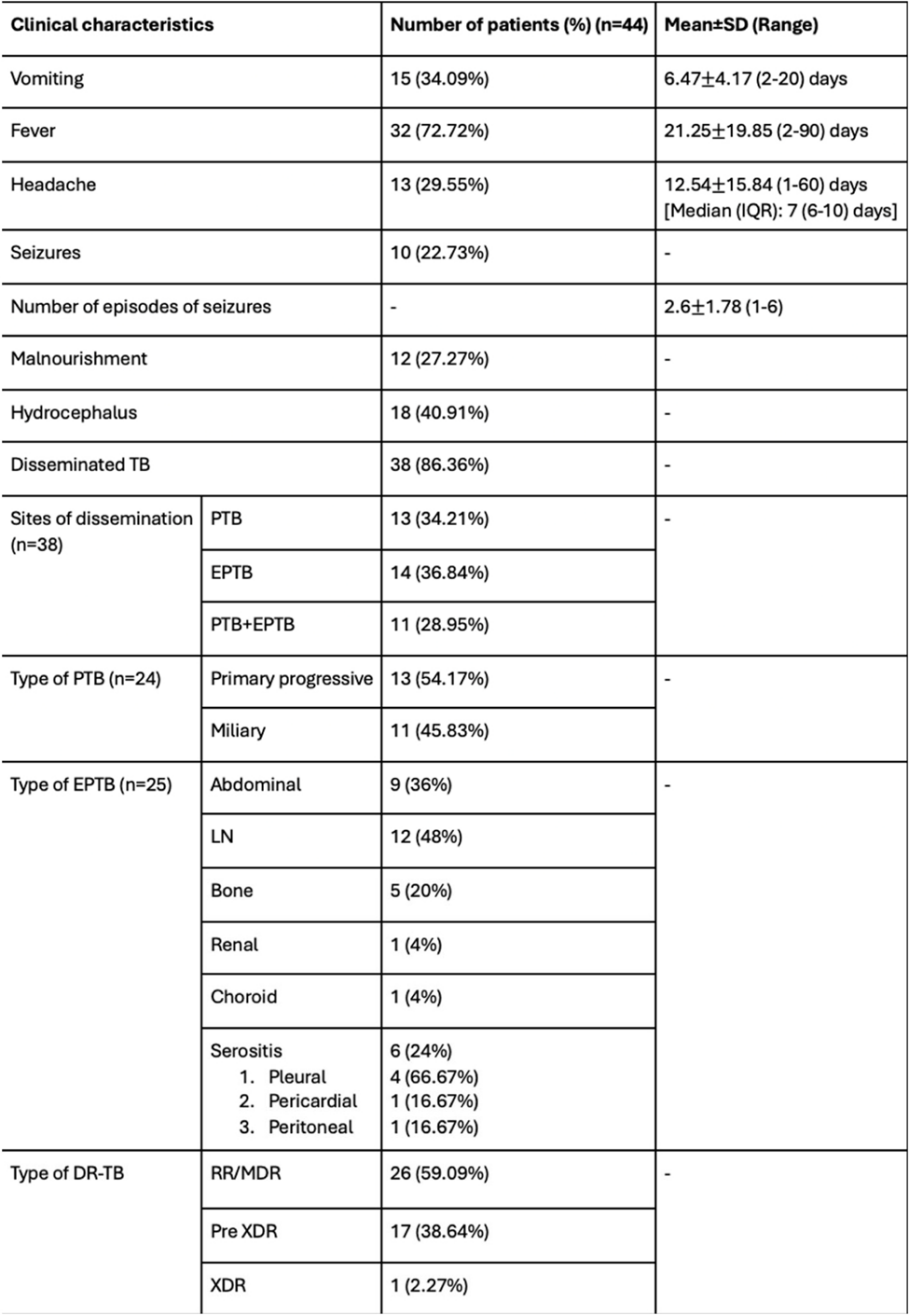
Clinical characteristics of the patients at presentation Note: SD- Standard deviation, IQR- interquartile range, TB- Tuberculosis, PTB- Pulmonary tuberculosis, EPTB- Extrapulmonary tuberculosis, LN- Lymph node, DR-TB- Drug-resistant tuberculosis, RR- Rifampicin resistant, MDR- Multidrug resistant, XDR- Extensively drug resistant.

**Table 2:**
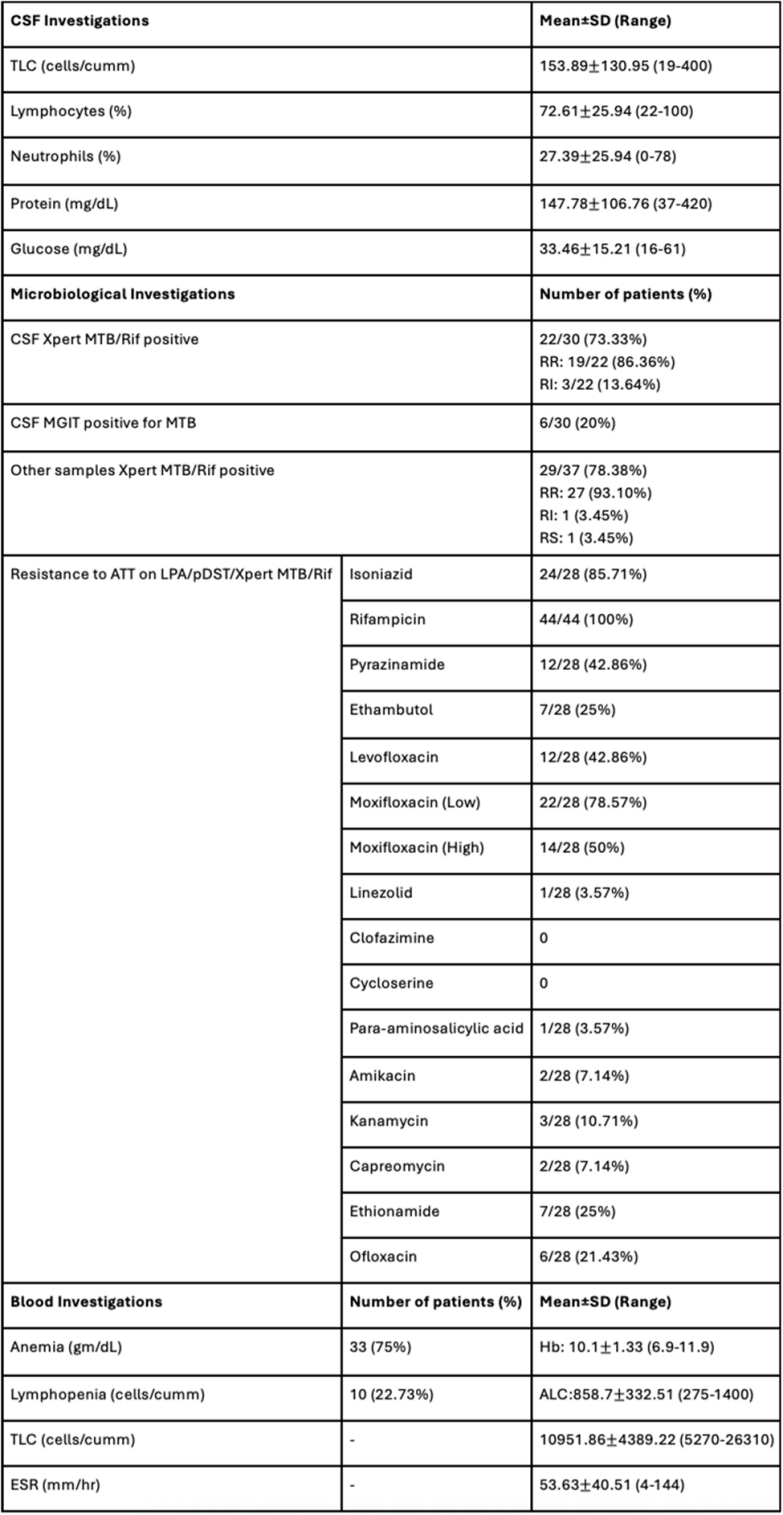
CSF, microbiological, and blood investigations of the patients at presentation Note: CSF- Cerebrospinal fluid, SD- Standard deviation, TLC- Total leukocyte count, RR- Rifampicin resistant, RI- Rifampicin indeterminate, RS- Rifampicin sensitive, MGIT- Mycobacterial Growth Indicator Tube, ATT- Antitubercular therapy, LPA- Line probe assay, pDST- Phenotypic drug sensitivity testing, Hb- Hemoglobin, ALC- Absolute lymphocyte count, ESR- Erythrocyte sedimentation rate.

**Methods:**

A retrospective study was conducted from May 2020 to April 2024 and included 44 children less than 18 years of age, diagnosed with DR CNS-TB, treated with second-line antitubercular therapy (ATT) regimens, and followed-up on an outpatient basis. Demographic, clinical, laboratory, treatment, ADR, and outcome data was collected and analysed.

**Table 3:**
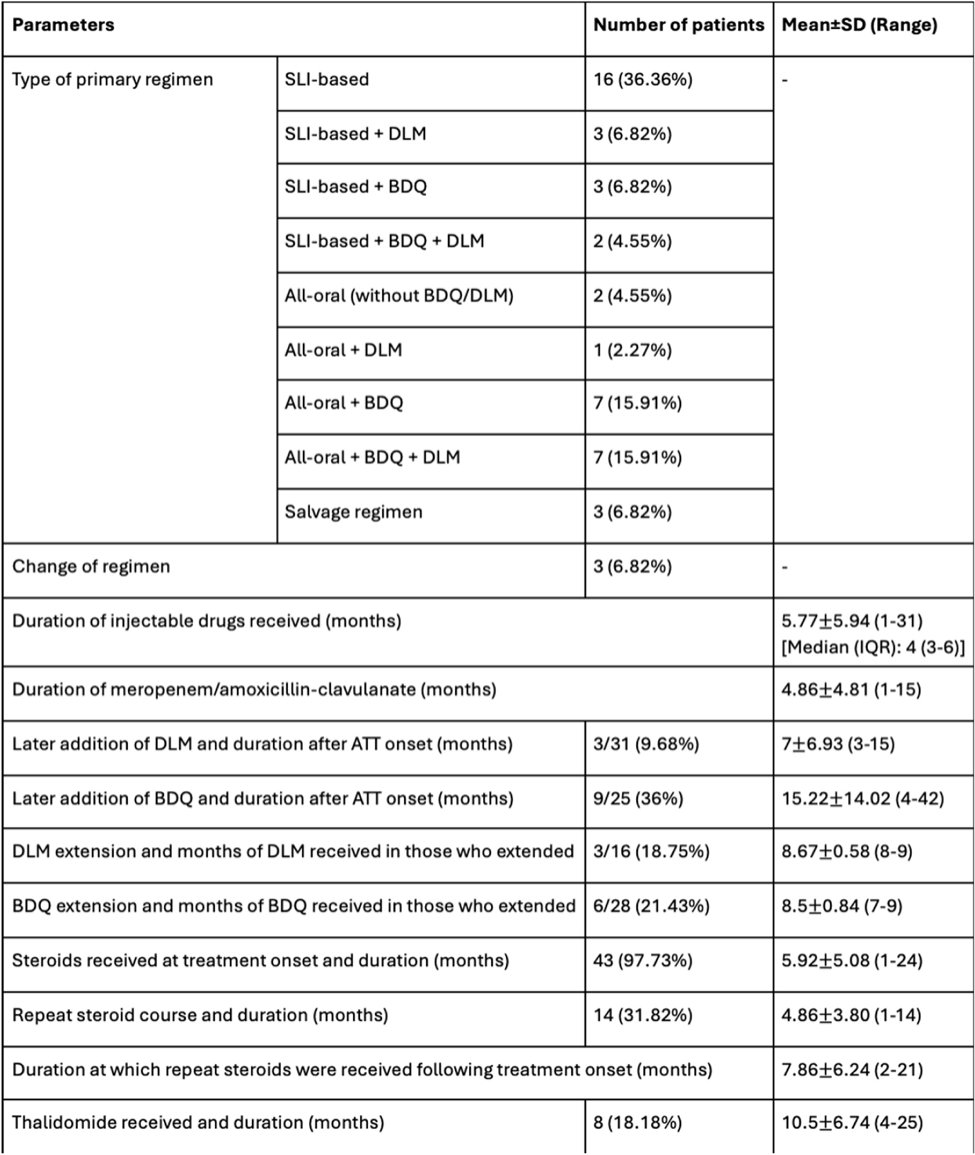
Treatment details of the patients Note: SD- Standard deviation, SLI- Second-line injectable, DLM- Delamanid, BDQ- Bedaquiline, IQR- Interquartile range, ATT- Antitubercular therapy.

**Table 4:**
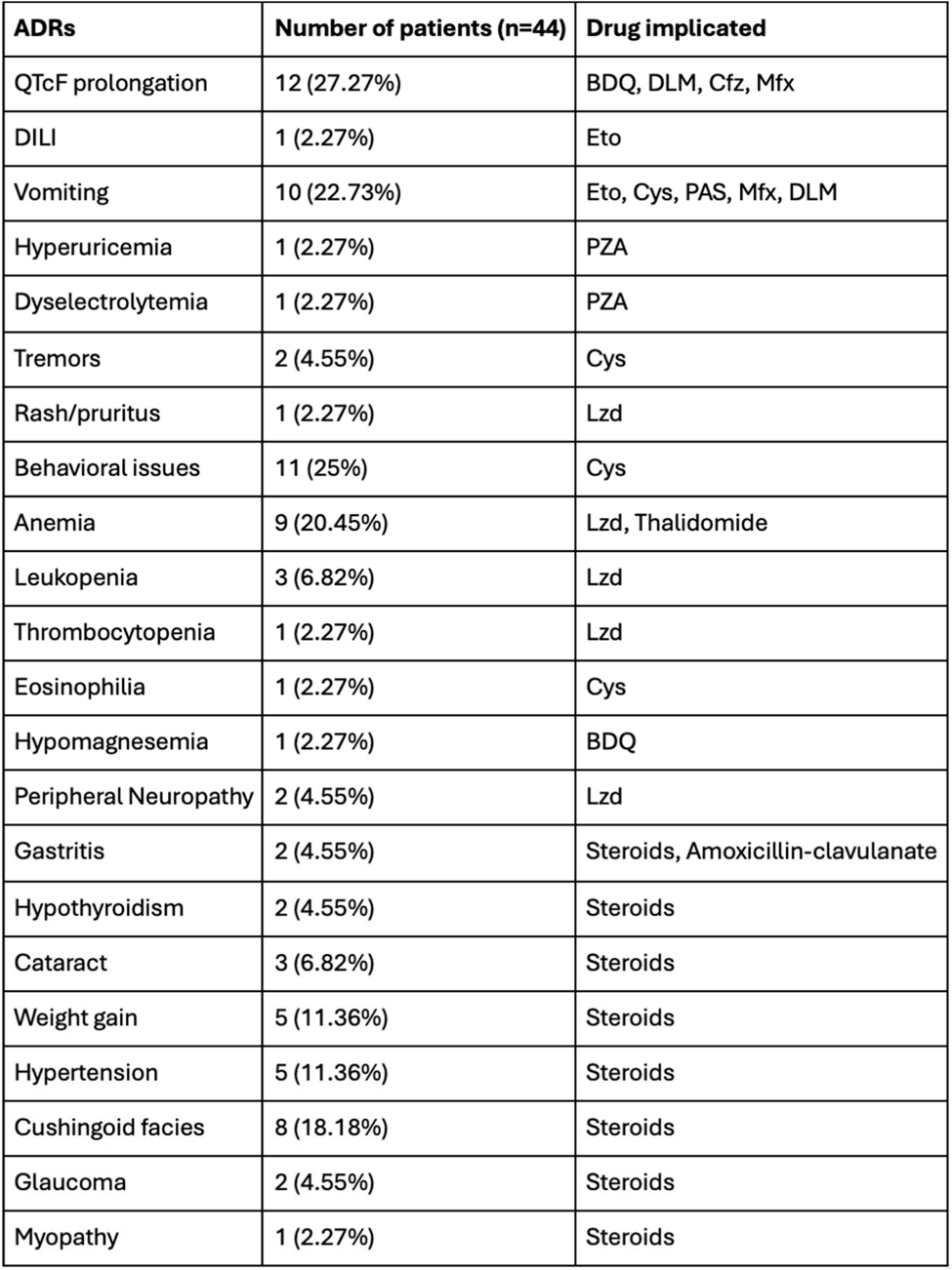
ADRs of antitubercular therapy, thalidomide and steroids Note: ADRs- adverse drug reactions, QTcF- QT interval corrected for heart rate by Fridericia's formula, BDQ- Bedaquiline, DLM- Delamanid, Cfz- Clofazimine, Mfx- Moxifloxacin, DILI- Drug-induced liver injury, Eto- Ethionamide, Cys- Cycloserine, PAS- Para-aminosalicylic acid, PZA- Pyrazinamide, Lzd- Linezolid.

**Results:**

Mean age at presentation was 9.67±4.41 years.Sixteen (36.36%) patients presented with TB meningitis alone, 7 (15.91%) patients presented with tuberculomas alone, and 21 (47.73%) presented with both. Past exposure to ATT was seen in 1 (2.27%) patient. Neurological deficits were observed in 16 (36.36%) patients during therapy, of which 4 (25%) had hemiparesis ± cranial nerve palsies, 1 (6.25%) had paraparesis with facial palsy, 1 (6.25%) had monoplegia with motor aphasia, and 10 (62.5%) patients had isolated cranial nerve palsies. Visual abnormalities during treatment were seen in 7 (15.91%) patients and sensorineural hearing loss (SNHL) was seen in 11 (25%) patients during treatment. New tuberculomas while on treatment were seen in 26 (59.09%) patients. ADR were seen in 36 (81.82%) patients. Mean duration of ATT given was 22.57±13.13 months. Of the 16 patients who developed motor deficits, 6 (37.5%) had residual motor deficits (1 had blindness, 6 had SNHL, 1 had residual hemiparesis and facial palsy, 3 had cranial nerve palsies), 3 (18.75%) recovered, 5 (31.25%) were still ongoing treatment, and 2 (12.5%) were lost to follow-up.

**Conclusion:**

Pediatric DR CNS-TB is difficult to treat with high rates of ADR, long treatment durations, and unfavourable neurological outcomes.

**Disclosures:**

All Authors: No reported disclosures

